# Supplementary material for: Structure and Properties of Electrochemically Synthesized Silver Nanoparticles in Aqueous Solution by High-Resolution Techniques
Source: Molecules. 2021 Aug 25;26(17):5155. doi: 10.3390/molecules26175155 (PMC8433840; doi:10.3390/molecules26175155)

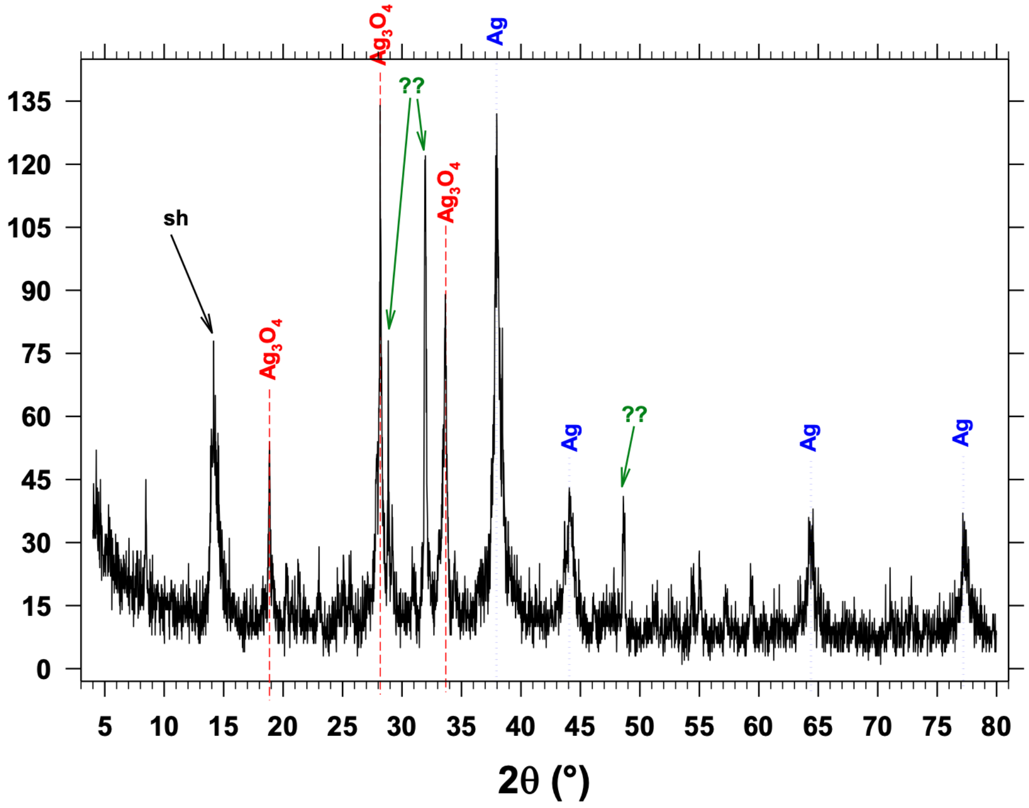

Figure S1. XRPD pattern and LeBail refined cell parameter for the investigated AgNPs. The peak marked with **sh** is due to the Si sample holder, while the peaks marked with **?** are due to undetermined other phases; however, some standard crystalline AgO compounds partially fit the peaks marked with **?**. The low amount of powders determined very low intensities of Bragg reflections.

Table S1. Refined LeBail cell parameters

LeBail refined cell parameters				
crystalline phase	space group	a	b	c
Ag metallic	Fm3m	4.099(1)	4.099(1)	4.099(1)
Ag_3O_4	$P2_1/c$	unrefined		

Table S2. XRPD peak Search Report

[2019_6152 SUNC_1.raw] SUNC 1-FT 0.02 deg-2.0 s								Peak Search report
SCAN	5.0/120.0/0.02/2(sec),Cu(45kV,40mA), I(max)=2545							
PEAK	29-pts/Parabolic Filter, Threshold=7.0, Cuoff=0.0%, BG=3/1.0, Peak-Top=Summit							
NOTE	Intensity=Counts, 2T(0)=0.0(°), Wavelength to Compute d-Spacing = 1.54056A (Cu/K-alpha 1)							
#	2-Theta	d(A)	BG	Height	I%	Area	I%	FWHM
1	27.818	3.2045	32	95	4.0	1400	2.9	0.251
2	32.203	2.7774	32	260	10.9	3029	6.2	0.198
3	38.101	2.3599	161	2384	100.0	49108	100.0	0.350
4	44.280	2.0439	107	692	29.0	17330	35.3	0.426
5	46.202	1.9632	30	130	5.5	2247	4.6	0.294
6	64.421	1.4451	71	480	20.1	12760	26.0	0.492
7	77.400	1.2320	94	469	19.7	14410	29.3	0.522
8	81.501	1.1800	44	153	6.4	4327	8.8	0.481
9	110.459	0.9377	73	151	6.3	4827	9.8	0.543
10	110.921	0.9351	73	105	4.4	5001	10.2	0.810
11	114.959	0.9135	74	158	6.6	5732	11.7	0.617

Table S3. MALDI mono isotopic masses used for calibration

MALDI parameters calibration									
K^+	CHCA $[M+H-H_2O]^+$	CHCA $[M+H]^+$	CHCA $[M+Na]^+$	CHCA $[2M+H-CO_2]^+$	CHCA $[2M+H]^+$	Bradykinin(1-7) $[M+H]^+$	Angiotensin II $[M+H]^+$	Instument setting	Pulsed ion extraction time
38.9637	172.0399	190.0504	212.0324	335.1032	379.0930	757.3991	1046.5418	19.00 kV and 16.70 kV	130 ns

Figure S2. Geometric correlation of synthesized SYNc shape with Silver XRD on online Database

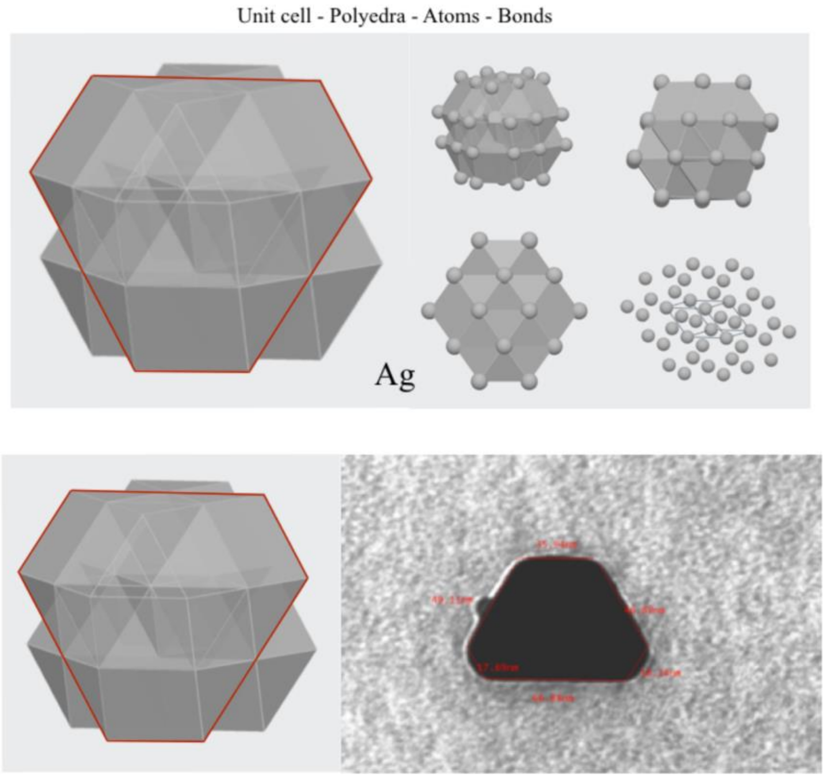

Supplement: Supplementary file 1 [file molecules-26-05155-s001.zip › molecules-1319714-suppl.pdf]
